# What Should be Taken into Consideration for a Meta-Analysis of Green Tea Consumption and Stomach Cancer Risk?

**DOI:** 10.4178/epih/e2010012

**Published:** 2010-12-31

**Authors:** Jeongseon Kim

**Affiliations:** Cancer Epidemiology Branch, Research Institute, National Cancer Center, Goyang, Korea.

Stomach cancer is the most common cancer in Japan, China and Korea, and is the second leading cause of deaths from cancer globally, although the incidence and mortality have been declining over the years [1]. Dietary factors are known to play an important role in the development of stomach cancer [2]. Among dietary factors, green tea has recently been of considerable interest in the etiology of stomach cancer [3]. It has been hypothesized that green tea consumption has a protective effect against the development of stomach cancer [4]. The main constituents of green tea extracts are polyphenols known as "catechins", which have been reported to have anticarcinogenic, antimutagenic and antioxidant activity in experimental studies using in vivo animal models [5]. An article was published by Kang et al. [6] on a meta-analysis of green tea consumption and its relation to stomach cancer. They did further subgroup analyses with a variety of effect sizes in order to explore the causes of heterogeneity among different studies. They also converted all the measured consumption levels of green tea to a cups-per-day scale to calculate the difference between the highest and lowest consumption levels. With such elaborate statistical approaches, their conclusion was that green tea may play a protective role against the development of stomach cancer. It is important to perform subgroup analyses since there are discrepancies in the effects of green tea consumption on stomach cancer risk between case-control studies and cohort studies as well as between crude data and adjusted data in the meta-analysis of published epidemiologic studies, for instance.

More interestingly, when stratified by country (Japan versus China) in the subgroup analyses performed by Kang et al. [6], a significant risk reduction by 39% (summary RR/OR=0.61, 95% CI: 0.47-0.81) was found with homogeneity (p=0.43) from the Chinese studies, while a non-significant risk reduction by 8% (summary RR/OR=0.92, 95% CI: 0.80-1.05) was observed with marginal homogeneity (p=0.10) from the Japanese studies. Part of the reason for the different results between the Chinese and Japanese studies could be that the seven cohort studies among the twelve Japanese studies failed to support the association (summary RR/OR=1.03, 95% CI: 0.92-1.16). Another part of the reason for the different results could be due to the varieties of green tea that undergo a type of roasting to dry the leaves in China [7]. These teas often characterize the Chinese varieties on the market, which provide a much more roasted flavor. In Japan, the green tea leaves are steam dried, creating a fresh grassy infusion. Many Japanese teas can be characterized by this technique and the light flavor that it produces. Since there have been no epidemiological studies investigating green tea consumption related to stomach cancer among a Korean population, which is one of biggest consumers in the world, it is limited to apply this meta-analytical result to Koreans with inferential interpretation. Hence, questions should be answered with more scientific evidence on whether the discrepancies may be due to either genetic background, environmental variables such as dietary factors, or the research methodology and design implemented.

It should be noted that there are some limitations regarding the interpretation of the meta-analysis. First, as green tea is consumed mainly in Asian countries, such as China, Japan, Korea, and a few countries in the Middle East and North Africa [8], we are not able to generalize our findings to all populations. Second, a random-effect model is usually selected in order to adjust for the effect of heterogeneity across studies but this model does not discount the effects of heterogeneity. In addition, another bias can occur in this meta-analysis. Publication bias can cause researchers to reach incorrect conclusions from their meta-analyses because studies with statistically significant results tend to be published. Although Begg's test shows that there is no significant publication bias in this meta-analysis, we can not ignore the possibility of this bias, inherent in any meta-analysis. Therefore, the conclusions or interpretations made from the results of this meta-analysis should be considered cautiously due to the above limitations and biases. The findings and explanations should be explored in future research, including clinical trials and further epidemiologic studies.
